# Synthesis of ZnO Nanoparticles From *Pergularia daemia* Fibre: Potential for Caries Prevention and Infection Control

**DOI:** 10.1016/j.identj.2025.109396

**Published:** 2026-01-28

**Authors:** Raja Thandavamoorthy, Yuvarajan Devarajan, Kulmani Mehar

**Affiliations:** a Materials Science Lab, Department of Prosthodontics, Saveetha Dental College and Hospitals, SIMATS, Chennai, Tamil Nadu, India; b Department of Mechanical Engineering, Saveetha School of Engineering, Saveetha Institute of Medical and Technical Sciences (SIMATS), Saveetha University, Chennai, 602105, Tamil Nadu, India; c Manipal Academy of Higher Education, Manipal Institute of Technology, Manipal, Karnataka, 576104, India

**Keywords:** ZnO nanoparticles, Dental applications, Biofilm inhibition, Antibacterial activity, Sustainable

## Abstract

**Introduction and aims:**

Antimicrobial resistance and biofilm-associated infections present major challenges in oral healthcare, necessitating sustainable nanomaterials with multifunctional efficacy. This study reports the green synthesis of zinc oxide nanoparticles (ZnO NPs) from *Pergularia daemia* (*P. daemia*) fibre (PDF) extracts and evaluates their structural, thermal, and biological properties for dental and biomedical applications.

**Methods:**

ZnO NPs were synthesised using aqueous PDF extracts as natural reducing and stabilising agents. Structural crystallinity was examined by X-ray diffraction (XRD), functional groups by Fourier-transform infrared spectroscopy (FTIR), morphology by scanning electron microscopy (SEM), and thermal stability by thermogravimetric analysis (TGA). Antibacterial performance against *Klebsiella pneumoniae* and *Streptococcus mutans* was assessed via agar well diffusion, while antibiofilm efficacy was evaluated using confocal laser scanning microscopy (CLSM).

**Results:**

XRD confirmed a semi-crystalline ZnO phase with a crystallite size of 28.6 nm and a crystallinity index of 21%. FTIR revealed hydroxyl, carbonyl, and carboxylate groups contributing to nanoparticle stabilisation. SEM micrographs showed irregular, porous, and agglomerated morphologies spanning nanometre to submicron scales. TGA indicated multi-step degradation with a stable residual fraction of ∼14% at 670 °C. Antibacterial assays demonstrated strong inhibition zones (27 mm, 32 mm at 75 µg; 31 mm, 41 mm at 100 µg), comparable to streptomycin (34 mm and 43 mm). CLSM confirmed significant antibiofilm activity through membrane disruption and reduced bacterial viability.

**Conclusion:**

The enhanced antibacterial and antibiofilm performance of PDF-derived ZnO NPs arises from synergistic effects of nanoparticle cores, reactive oxygen species generation, and phytochemical surface functionalization. Their stability and bioactivity underscore their promise as sustainable nanomaterials.

**Clinical relevance:**

PDF-mediated ZnO NPs show potential for dental applications, including caries prevention, root canal disinfection, and biofilm-resistant coatings for restorative and implant materials. Their multifunctional profile further supports broader biomedical use in antimicrobial therapy, drug delivery, and composite engineering.

## Highlights

• Green synthesis of zinc oxide (ZnO) nanoparticles using *Pergularia daemia* (*P. daemia*) stem fibres as a sustainable bio-resource.
• Phytochemical-assisted reduction and stabilisation achieved without toxic chemicals.
• Formation of semi-crystalline ZnO nanoparticles confirmed by X-ray diffraction with JCPDS comparison.
• Porous and irregular nanoparticle morphology revealed by scanning electron microscopy, enhancing surface activity.
• Strong antibacterial and antibiofilm efficacy against *Klebsiella pneumoniae* and *Streptococcus mutans* was demonstrated.
• Potential application of biosynthesised ZnO nanoparticles in caries prevention and oral infection control.

## Graphical abstract

**Figure:**
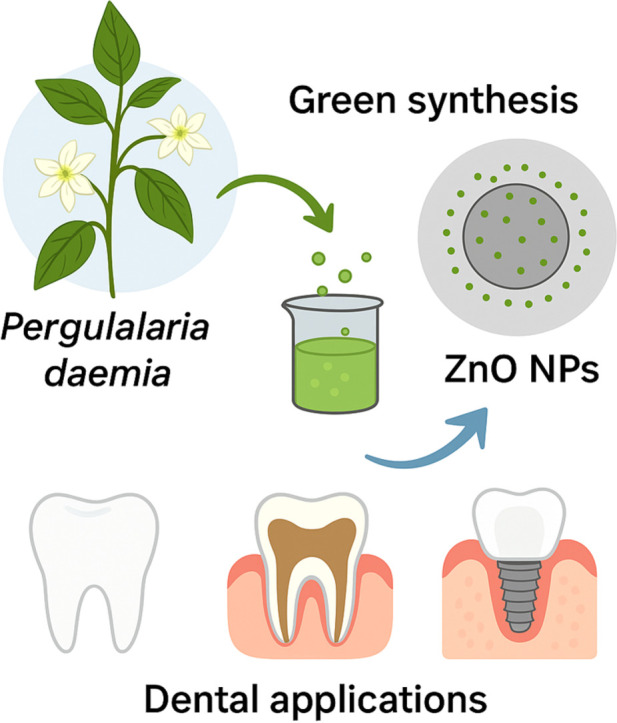

## Introduction

The rapid expansion of nanotechnology has driven a search for sustainable and eco-friendly synthesis routes for metal and metal oxide nanoparticles. Conventional methods such as sol–gel, hydrothermal, and chemical precipitation often require toxic precursors, surfactants, or high-energy inputs, raising environmental and biomedical safety concerns.^1^ In response, green synthesis has emerged as a promising alternative that employs biological systems, particularly plants, as reducing and stabilising agents in nanoparticle fabrication.^2^ This approach is not only environmentally benign but also cost-effective and compatible with large-scale applications, aligning with the principles of green chemistry and circular bioeconomy. Plant-mediated nanoparticle synthesis is particularly attractive because plant tissues are rich in secondary metabolites such as flavonoids, phenolics, alkaloids, tannins, terpenoids, and saponins. These compounds act synergistically as reducing agents, stabilisers, and capping molecules, enabling controlled nucleation and growth of nanoparticles.^3^

Several studies have demonstrated the efficacy of this approach. For instance, banana peel and stem extracts have been employed in the biosynthesis of ZnO and Ag nanoparticles, showing enhanced antibacterial and antioxidant activities.^4^ Similarly, jute fibres and hemp extracts have been explored for ZnO NP formation, yielding structures with excellent antimicrobial and UV-protective properties.^5^ Sisal and coir fibres, owing to their lignocellulosic matrices, have also been successfully applied in nanoparticle synthesis, confirming the potential of natural fibres as biogenic sources.^6^ Green-synthesised ZnO NPs, in particular, have gained attention for their multifunctionality. Beyond their recognised photocatalytic activity in wastewater treatment,^7^ ZnO NPs possess significant biomedical potential due to their ability to generate reactive oxygen species (ROS), disrupt bacterial membranes, and interfere with intracellular biomolecules.^8^ Recent reports also highlight their antibiofilm efficacy, where their nanoscale dimensions and phytochemical coatings facilitate penetration into extracellular polymeric substances, leading to disintegration of microbial colonies.^9^

Furthermore, studies have confirmed that biogenic ZnO NPs often exhibit improved colloidal stability and biocompatibility compared to chemically synthesised analogues, owing to the organic functional groups from phytochemicals adsorbed on their surfaces.^10^ Despite the growing body of work on plant-mediated ZnO NPs, relatively few studies have investigated the potential of *P. daemia* as a nanoparticle precursor. *P. daemia* is a medicinally significant plant in the Asclepiadaceae family, traditionally used for its antimicrobial, anti-inflammatory, and wound-healing properties.^11^ Its stem fibres exhibit a tensile strength of 18.5 to 25 MPa, elongation at break of 1.8% to 2.3%, low density (∼1.26 g/cm³), and thermal stability up to 334 °C.^12^ Chemically, the fibres contain 58% to 60% cellulose, 21% hemicellulose, and ∼15% lignin, providing abundant hydroxyl and carbonyl groups suitable for reducing metal salts into nanoparticles. The porous, rough surface morphology enhances interfacial interactions, while its bioactive phytochemical profile makes it a strong candidate for biosynthetic nanoparticle stabilisation. However, to date, there has been minimal exploration of its potential in green nanotechnology, making it a novel and underutilised resource. Natural fibres have long been recognised as sustainable reinforcements for composites due to their low density, biodegradability, renewability, and relatively high specific strength. Fibres such as jute (*Corchorus olitorius*), flax (*Linum usitatissimum*), hemp (*Cannabis sativa* L), banana (*Musa acuminata*), coir (*Cocos nucifera*), and sisal (*Agave sisalana*) are increasingly being integrated into polymer matrices to produce lightweight structural composites.^13^ Their lignocellulosic composition offers not only mechanical strength but also a surface chemistry conducive to modification and functionalisation. When hybridised with nanoparticles, these fibres can yield multifunctional composites with improved thermal stability, flame retardancy, and antimicrobial performance.^14^

The incorporation of nanoparticles such as ZnO, TiO₂, SiO₂, or Al₂O₃ into natural fibre composites has been shown to significantly enhance tensile, flexural, and impact strength while reducing water absorption and improving dimensional stability.^15^ This synergy between natural fibres and nanoparticles provides a sustainable pathway for the development of advanced bio-nanocomposites. In recent years, these hybrid systems have found growing applications in the automotive and transportation sectors. Natural fibre–reinforced composites have been successfully utilised in door panels, dashboards, seat backs, and trunk liners, where light weighting contributes to fuel efficiency and reduced carbon emissions. The addition of nanoparticles further enhances wear resistance, acoustic insulation, and thermal properties, enabling their deployment in more demanding automotive environments. For example, ZnO and clay nanoparticles integrated with flax or jute fibres have been reported to improve flame retardancy and UV stability, critical for automotive interior parts.^16^ Similarly, banana and sisal fibre composites reinforced with metal oxide nanoparticles are being investigated for under-the-hood components, owing to their superior thermal resistance. Beyond automotive uses, such natural fibre–nanoparticle composites are also gaining traction in aerospace lightweight structures, marine applications, and eco-friendly packaging solutions, where the demand for sustainable yet high-performance materials is rapidly increasing.^17^

*Problem statement:* Oral infections such as dental caries and biofilm-associated diseases are increasingly difficult to manage due to rising antimicrobial resistance and the limited long-term efficacy of conventional chemical agents. Although ZnO NPs show strong antimicrobial potential, most synthesis routes rely on toxic chemicals, high energy consumption, and non-sustainable processes, limiting their biomedical translation. Furthermore, underutilised plant fibres remain largely unexplored as green resources for nanoparticle fabrication. Therefore, there is a clear need for an eco-friendly, biocompatible, and resource-efficient approach to synthesise functionally stable ZnO NPs suitable for oral healthcare applications.

Unlike most green ZnO NP syntheses that rely on widely explored plant sources such as banana stem, jute, or neem, the present study investigates *P. daemia* stem fibres as an underexplored and non-conventional bioresource for nanoparticle fabrication. The key research gap addressed is the absence of fibre-based green synthesis strategies capable of simultaneously providing effective phytochemical-driven stabilisation, porous surface morphology, and strong antibiofilm activity relevant to oral healthcare applications. The novelty of this work lies in the distinctive phytochemical composition of *P. daemia* fibres, which enables their dual role as natural reducing and capping agents, leading to the formation of semi-crystalline, organically capped, and highly surface-active ZnO NPs. Accordingly, the aim of this study was to develop a sustainable, plant-mediated route for the synthesis of ZnO NPs using *P. daemia* fibre extracts and to systematically investigate their structural, thermal, antibacterial, and antibiofilm properties. Specifically, this work seeks to (1) valorise *P. daemia* fibres as an underutilised bioresource for green nanofabrication, (2) elucidate the role of fibre-derived phytochemicals in nanoparticle formation and stability, and (3) evaluate the suitability of the synthesised ZnO NPs for dental applications, including oral biofilm control, caries prevention, root canal disinfection, and antimicrobial surface coatings.

## Materials and experimental process

### Materials used

*P. daemia* stem fibres were collected from mature, healthy plants growing in Periyagaram village, Tiruvannamalai district, Tamil Nadu, India. The fibres were extracted using a water retting process, in which the stems were immersed in clean water for a controlled period to facilitate microbial degradation of non-fibrous tissues and enable easy separation of the fibres. The extracted fibres were thoroughly washed with distilled water and shade-dried at room temperature. Zinc acetate dihydrate (analytical grade, ≥ 99% purity) was used as the zinc precursor without further purification. All chemicals were of analytical grade, and deionised water was used throughout the experimental procedures to ensure consistency and reproducibility.

#### Green synthesis of nanoparticles from *P. daemia* fibres

**Fig. 1:**
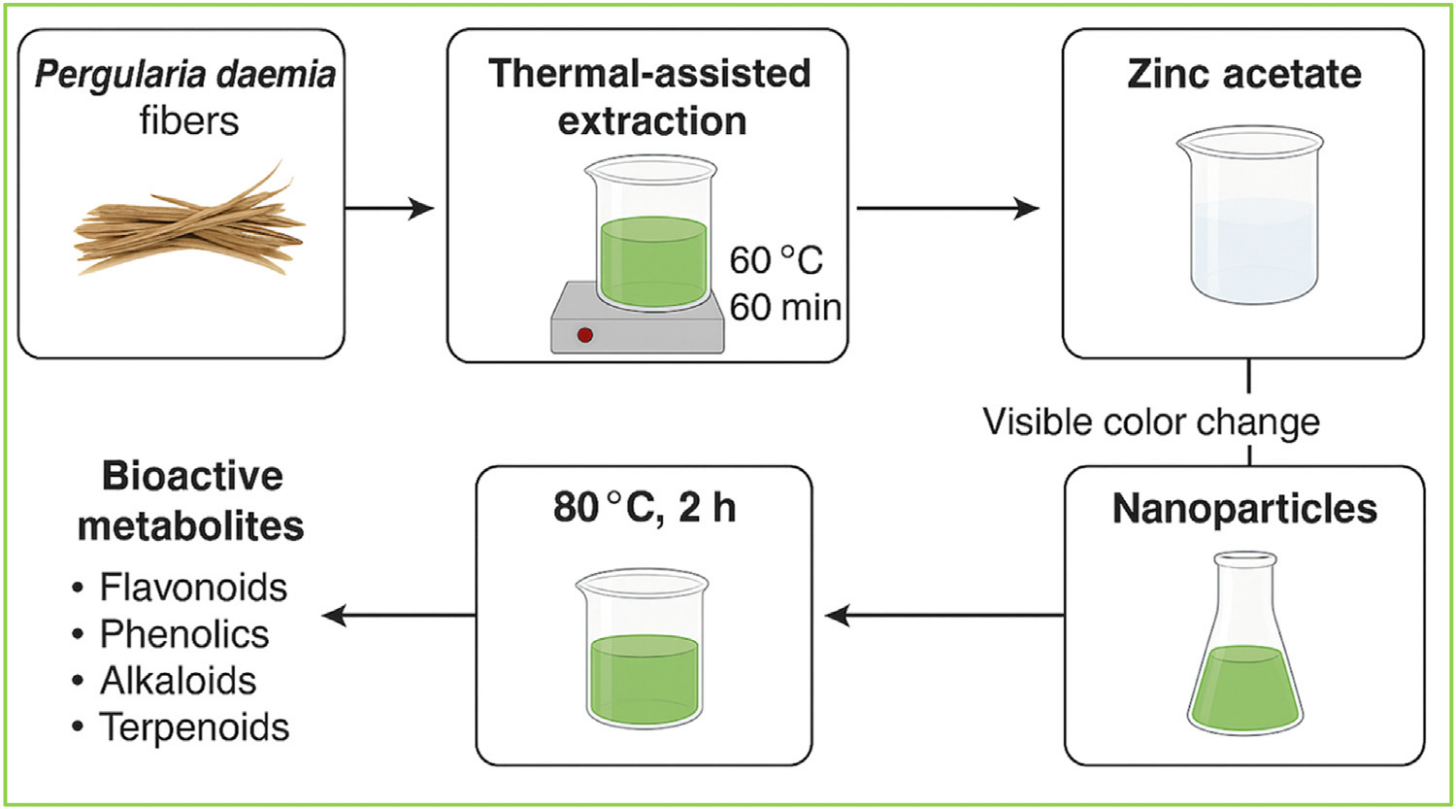
Green synthesis of nanoparticles from *P. daemia* fibres.

In this approach, aqueous extracts of the stem fibres are prepared by thermal-assisted extraction, wherein the fibrous biomass is incubated at 60 °C for 60 minutes. This process facilitates the liberation of diverse bioactive metabolites, including flavonoids, phenolics, alkaloids, tannins, and terpenoids, which play a pivotal role in nanoparticle nucleation and stabilisation. Upon mixing the prepared plant extract with the zinc precursor solution and maintaining continuous agitation at 80 °C, a gradual change in the solution colour becomes evident within 2 hours, indicating the initiation of ZnO NP formation. The aqueous extract was combined with a 0.1 mol/L zinc nitrate solution, and the reaction pH was carefully adjusted to 10 ± 0.2 using 1 mol/L NaOH to promote controlled nucleation and uniform particle growth. This chromatic transformation arises from optical absorption related to electronic transitions and defect states in ZnO NPs, confirming the phytochemical-mediated reduction of metal ions and successful nanoparticle formation. At the molecular level, polyphenolic compounds and hydroxyl-rich flavonoids act as potent electron donors, driving the reduction of Zn²⁺ ions, while functional moieties such as carboxyl, hydroxyl, and carbonyl groups simultaneously coordinate with nascent nuclei to prevent uncontrolled agglomeration.^18^ This dual functionality not only facilitates the reduction process but also imparts colloidal stability through capping and steric hindrance. The stabilisation effect is further reinforced by hydrogen bonding and van der Waals interactions contributed by alkaloids and other phytoconstituents in the extract. After completion of the reaction, the nanoparticle suspension was filtered using Whatman No. 1 filter paper, and the collected solid was washed three times with distilled water and ethanol to remove unreacted phytochemicals. The filtrate was then oven-dried at 60 °C for 12 hours to obtain a stable dry powder. Finally, the dried precipitate was calcined at 450 °C for 2 hours in a muffle furnace, a critical step to ensure complete decomposition of organic residues and the formation of crystalline ZnO NPs.^19^ Unlike conventional chemical methods that employ sodium borohydride or hydrazine, the phytogenic approach is inherently non-toxic, cost-effective, and energy-efficient, thereby aligning with green chemistry principles and circular bioeconomy practices. The as-synthesised nanoparticles exhibit notable colloidal stability, attributed to the persistent phytochemical capping, which minimises particle coalescence and enhances dispersion in aqueous media. Such stability is advantageous for downstream applications in biomedical, dental, and material science domains, where nanoparticle aggregation often undermines efficacy. Thus, the green synthesis of nanoparticles from *P. daemia* fibres exemplifies a phytochemical-driven, eco-benign nanofabrication strategy in which naturally occurring metabolites act synergistically as reducing, capping, and stabilising agents. This bioinspired route not only advances the production of functionally stable nanomaterials but also contributes to sustainable technological development by valorising agricultural biomass into high-value nanostructures. Figure 1 shows the green synthesis of nanoparticles from *P. daemia* fibres.

### Experimental methods

The synthesised *P. daemia* nanoparticles were subjected to a series of structural, chemical, thermal, and biological tests in accordance with standardised protocols.

#### Characterisation of ZnO NPs

X-ray diffraction (XRD) analysis was performed using a Rigaku Ultima IV diffractometer equipped with Cu-Kα radiation (λ = 1.5406 Å). The nanoparticles were pressed into flat glass holders and scanned over a 2θ range of 10-80° at a scan rate of 2°/min. Fourier Transform Infrared Spectroscopy (FTIR) was carried out using a Bruker Alpha II spectrometer. Samples were prepared by mixing 2 mg of nanoparticles with 200 mg of dried KBr powder, pressing into pellets of 13 mm diameter, and scanning in the range of 4000 to 400 cm⁻¹ with a resolution of 4 cm^−^¹. SEM and EDX analysis were performed using a JEOL JSM-IT300. Powdered nanoparticle samples were mounted on aluminium stubs with carbon adhesive tape and sputter-coated with a 10 nm gold layer using a Quorum Q150R ES coater. Imaging was carried out at an accelerating voltage of 15 kV to study surface morphology.^20^ Thermogravimetric analysis (TGA/DTG) was conducted using a PerkinElmer STA 6000 simultaneous thermal analyser. A 10 mg of nanoparticle powder was placed in a platinum crucible and heated from 30 to 900 °C at a constant rate of 10 °C/min under a nitrogen flow of 40 mL/min. ASTM E1131 standard guidelines were followed to ensure reproducibility of thermal data.^21^ All experimental tests were carried out in triplicate to ensure consistency and reliability of results.

#### Biological activity

Antibacterial testing was conducted using the agar well diffusion method following CLSI guidelines. The Gram-negative pathogen *Klebsiella pneumoniae* (ATCC 13883) and the Gram-positive oral bacterium *Streptococcus mutans* (ATCC 25175) were obtained from the Microbial Type Culture Collection (MTCC), Chandigarh, India, an accredited national repository. The agar well diffusion assay was performed using Mueller–Hinton agar as the standard growth medium for evaluating the antibacterial activity of *P. daemia*–derived ZnO NPs against *K pneumoniae* and *S mutans.* Each well was filled with 50 μL of nanoparticle suspensions at concentrations of 75 and 100 μg/mL. Plates were incubated at 37 °C for 24 hours against *K pneumoniae* and *S mutans* bacterial cultures. The zone of clearance around each well was measured in millimetres using a Vernier caliper.^22^ Antibiofilm analysis was performed by confocal laser scanning microscopy (CLSM, Leica TCS SP8) using dual fluorescent staining with acridine orange (AO, 5 μg/mL) and propidium iodide (PI, 10 μg/mL). Biofilms were developed on sterile glass coverslips (10 × 10 mm) placed in 12-well plates containing Luria–Bertani broth inoculated with *K pneumoniae* and incubated at 37 °C for 48 hours. Following biofilm establishment, coverslips were treated with nanoparticle suspensions at 50 and 100 μg/mL for 6 hours before staining. The biofilm detection method used in this study was CLSM coupled with fluorescent live/dead staining. This approach is primarily qualitative, as it enables direct visualisation of biofilm architecture, thickness, and cell viability through differential fluorescence.^23^

## Results and discussion

### XRD analysis of *P. daemia* nanoparticles

The XRD pattern of *P. daemia* fibre-mediated nanoparticles reveals a series of sharp, well-resolved diffraction peaks, confirming the successful formation of crystalline zinc oxide with a hexagonal wurtzite structure. The prominent reflections located at 2θ values of 31.7°, 34.4°, 36.4°, 47.7°, 56.7°, and 62.9° can be indexed to the (100), (002), (101), (102), (110), and (103) crystallographic planes of ZnO, respectively, in close agreement with the standard JCPDS card No. 36-1451. The presence of additional higher-order reflections further confirms the high degree of crystallographic ordering and phase purity, while the absence of secondary peaks indicates that no zinc hydroxide or other zinc-containing impurity phases were formed during synthesis. Crystallite size analysis was performed using the Debye–Scherrer equation applied to the most intense diffraction peaks. The estimated crystallite sizes corresponding to the (100), (002), and (101) planes were 24.8 nm, 26.1 nm, and 29.4 nm, respectively. Slightly smaller crystallite sizes, in the range of 21 to 23 nm, were observed for higher-index planes such as (102) and (110). This plane-dependent variation in crystallite size suggests anisotropic crystal growth, which is characteristic of wurtzite ZnO due to its polar crystal structure. Preferential growth along the (101) plane, evidenced by its higher intensity and larger crystallite size, reflects its lower surface energy and greater thermodynamic stability under the present synthesis conditions. The average crystallite size, calculated from multiple diffraction planes, was 26.5 ± 2.8 nm, confirming the nanocrystalline nature of the synthesised ZnO. Minor peak broadening observed at higher diffraction angles can be attributed to a combination of lattice strain, finite crystallite size effects, and surface defect formation. Such microstructural distortions are commonly observed in phytochemical-assisted synthesis routes, where organic molecules partially interact with the growing crystal facets.

**Fig. 2:**
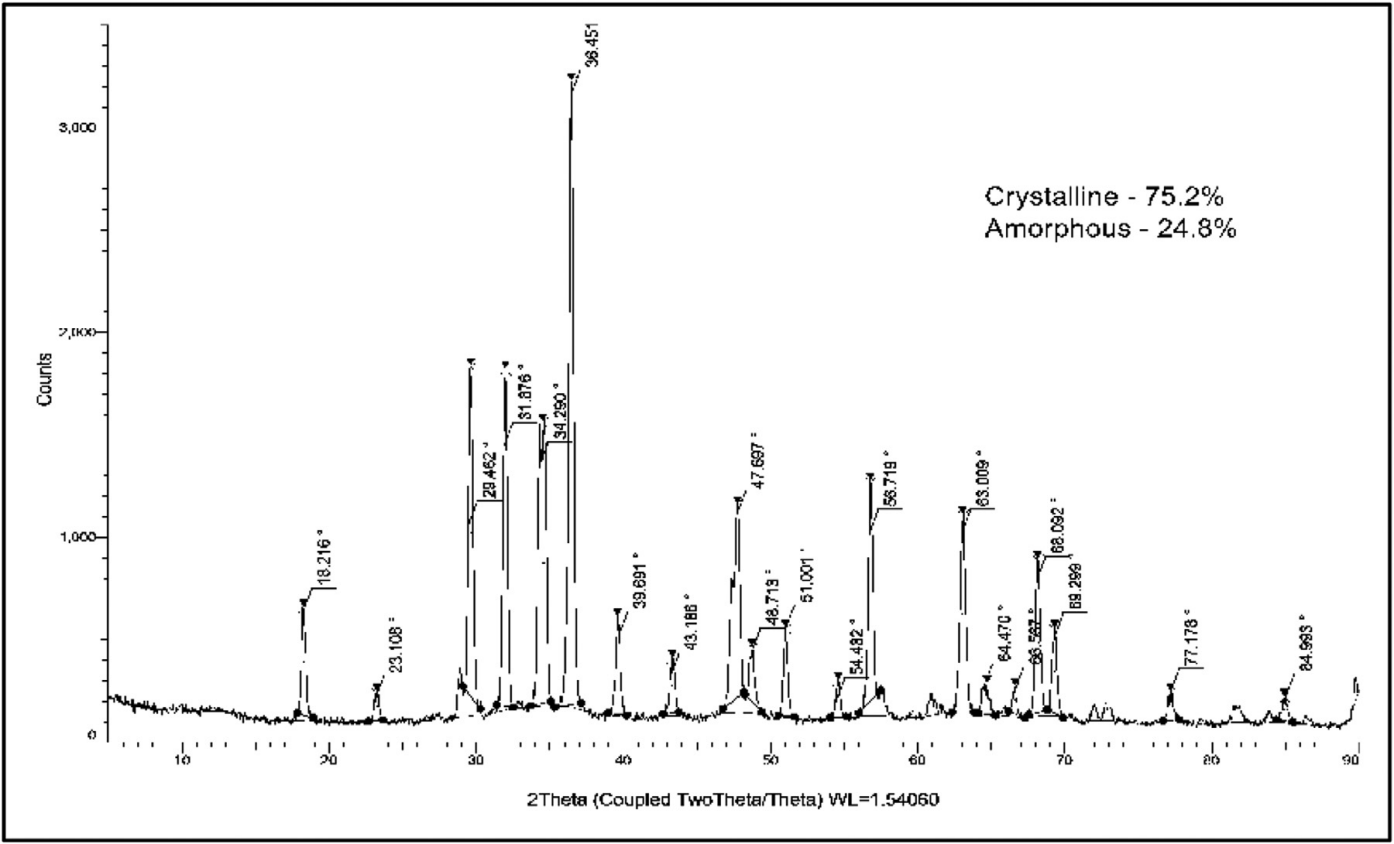
XRD curve of PDF nanoparticles.

Quantitative phase analysis indicates that the nanoparticles consist of 75.2% crystalline phase and 24.8% amorphous content. The residual amorphous fraction likely originates from organic moieties derived from *P. daemia* fibres, including cellulose fragments and polyphenolic residues, which remain weakly bound to the ZnO surface. These amorphous regions are predominantly localised at grain boundaries and surface layers, where they interfere with complete lattice periodicity but do not disrupt the overall ZnO crystal framework. From a mechanistic perspective, the green synthesis process involves rapid nucleation of ZnO nanocrystals facilitated by phytochemicals acting as reducing agents for Zn²⁺ ions. Subsequently, controlled crystal growth occurs as these biomolecules adsorb selectively onto specific crystallographic planes, moderating growth rates and preventing uncontrolled agglomeration. This selective adsorption leads to facet-dependent growth kinetics, resulting in well-defined crystallites with preserved nanoscale dimensions and moderate lattice strain. The coexistence of crystalline and amorphous phases plays a critical functional role. The crystalline ZnO domains provide mechanical stability, thermal robustness, and electronic band structure integrity, while the amorphous fraction introduces defect-rich active sites, oxygen vacancies, and increased surface energy. These features are particularly beneficial for applications such as antimicrobial activity, photocatalysis, and biomedical coatings, where surface reactivity and defect-mediated interactions are essential. Therefore, the XRD analysis confirms that *P. daemia*-derived nanoparticles are phase-pure, nanocrystalline ZnO with a hexagonal wurtzite structure and an average crystallite size of 26.5 nm. The plane-dependent crystallite growth and controlled amorphous content reflect the effectiveness of the phytochemical-assisted synthesis route in tailoring the structural characteristics of ZnO NPs for advanced functional applications. Figure 2 shows the XRD curve of PDF nanoparticles.

### FTIR spectrum of *P. daemia* nanoparticles

The FTIR spectrum of *P. daemia* fibre-derived ZnO NPs reveals a complex set of absorption bands that reflect the presence of phytochemical functional groups involved in nanoparticle formation, surface functionalization, and stabilisation during the green synthesis process. A broad absorption band centred around 3334 cm^−^¹ is attributed to O–H stretching vibrations of hydroxyl groups arising from phenolic compounds, alcohols, and polyhydroxylated plant metabolites. These hydroxyl functionalities play a key role in the reduction of Zn²⁺ ions by facilitating electron transfer and subsequently stabilising the nanoparticle surface through extensive hydrogen bonding networks, thereby suppressing uncontrolled particle agglomeration. The absorption band observed near 2913 cm^−^¹ corresponds to aliphatic C–H stretching vibrations of methylene groups, indicating the adsorption of plant-derived organic chains on the ZnO surface. Such hydrocarbon moieties contribute to surface passivation and influence interparticle interactions by providing steric hindrance. In the mid-infrared region, bands located at 1650.3 cm^−^¹ and 1603.3 cm^−^¹ are associated with carbonyl stretching vibrations of conjugated functional groups and C=C stretching of aromatic rings, respectively. These features confirm the involvement of polyphenols, flavonoids, and tannin-like compounds, which possess conjugated π-electron systems capable of coordinating with surface Zn atoms while simultaneously acting as reducing agents during nucleation. Additional absorption bands at 1506.7 cm^−^¹ and 1474.6 cm^−^¹ are assigned to N–H bending and C–N stretching vibrations, suggesting the presence of proteinaceous or peptide-derived residues. These nitrogen-containing groups can bind to ZnO surfaces through coordination interactions, enhancing steric stabilisation and contributing to the formation of a bio-organic shell around the inorganic core. The band observed at 1360.7 cm^−^¹ is attributed to symmetric stretching of carboxylate groups, indicating the presence of organic acids that impart electrostatic stabilisation and improve the dispersion stability of the nanoparticles in aqueous environments.

**Fig. 3:**
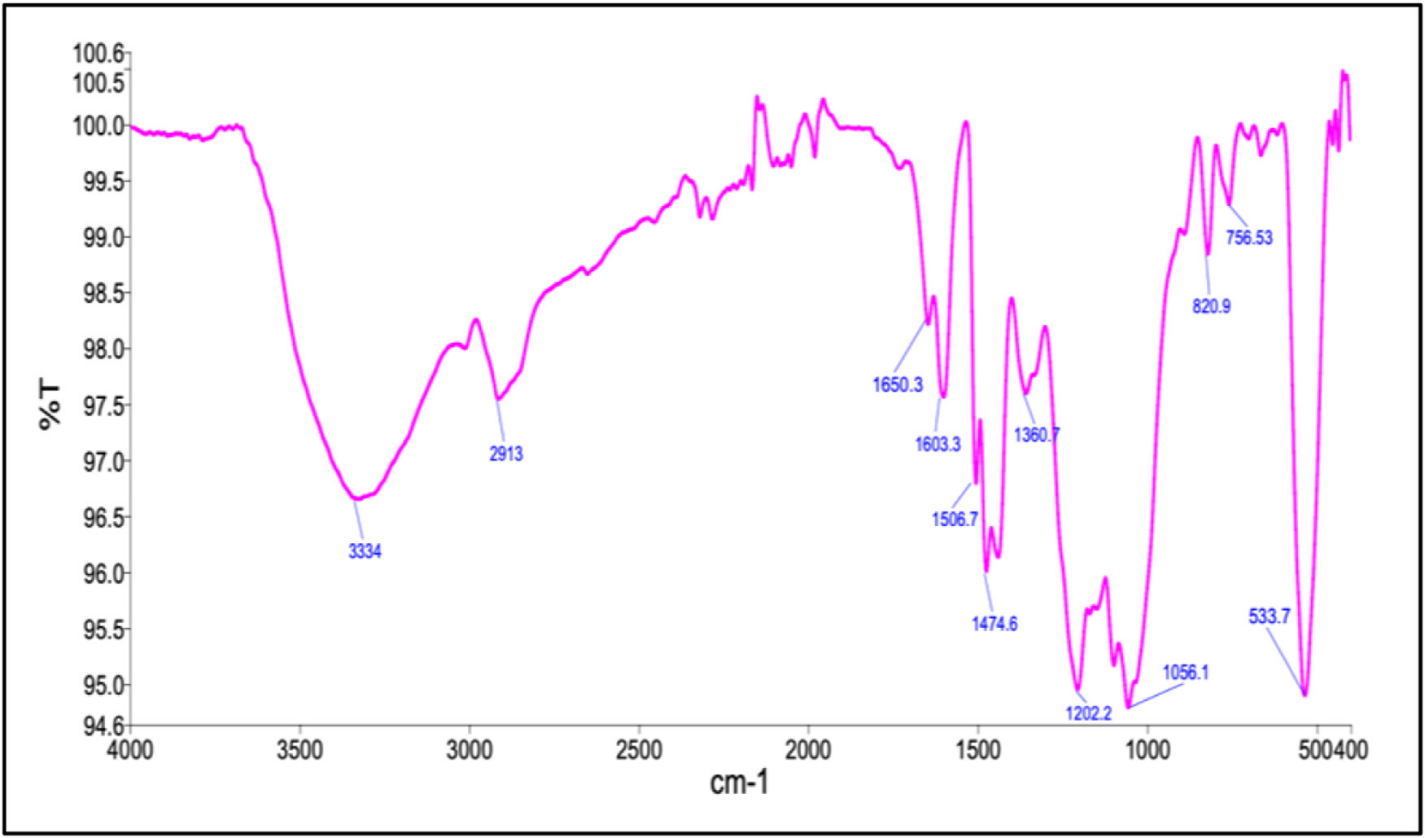
The FTIR spectrum of PDF nanoparticles.

In the lower wavenumber region, the absorptions at 1202.2 cm^−^¹ and 1056.1 cm^−^¹ are characteristic of ether linkages and C–O stretching vibrations of primary alcohols, confirming the presence of polysaccharides and glycosidic components derived from the plant fibre. These macromolecules function as natural capping agents, regulating crystal growth kinetics and restricting excessive particle coalescence. Bands observed at 820.9 cm^−^¹ and 756.5 cm^−^¹ correspond to out-of-plane bending vibrations of aromatic C–H bonds, indicating the contribution of lignin-derived or substituted aromatic structures associated with the nanoparticle surface. The most diagnostically important feature of the spectrum is the absorption band at 533.7 cm^−^¹, which is assigned to Zn–O stretching vibrations and provides direct confirmation of zinc oxide formation. The coexistence of this metal–oxygen vibration with numerous organic functional group absorptions demonstrates that the ZnO NPs are not bare but are instead surface-functionalised by phytochemical residues from *P. daemia* fibres. From a mechanistic perspective, the FTIR results suggest a synergistic role of multiple functional groups in the green synthesis process. Hydroxyl and carbonyl groups initiate the reduction of Zn²⁺ ions and promote nucleation, while amide, carboxylate, ether, and polysaccharide functionalities stabilise the growing ZnO nuclei through coordination, hydrogen bonding, and steric effects. This cooperative interaction leads to the formation of ZnO NPs encapsulated within a thin organic matrix, which enhances colloidal stability, introduces surface defects, and increases chemical reactivity. Therefore, the FTIR analysis confirms that the phytochemical-assisted synthesis route yields ZnO NPs with a hybrid organic–inorganic surface architecture. Such functionalisation is advantageous for applications requiring strong surface interactions and biological activity, including antimicrobial systems, biomedical coatings, biosensors, polymer nanocomposites, and environmental remediation technologies. Figure 3 shows the FTIR spectrum of PDF nanoparticles.

### SEM and EDX analysis of *P. daemia* nanoparticles

**Fig. 4:**
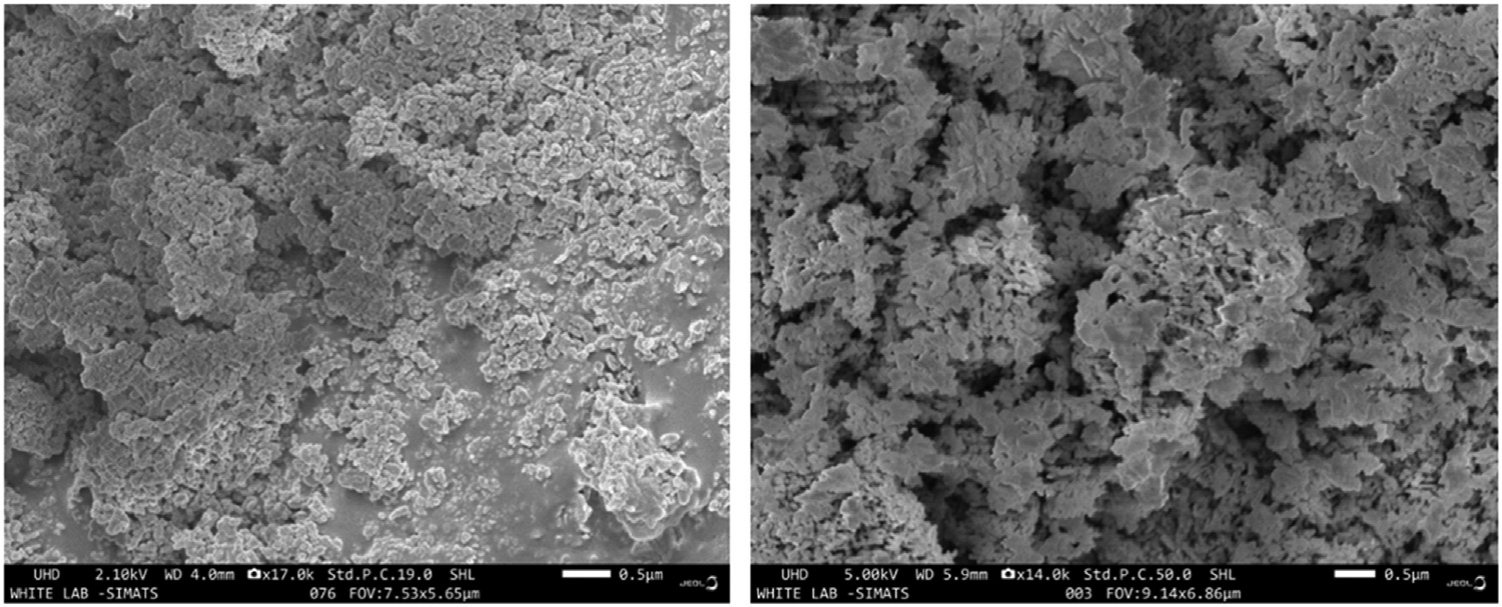
SEM microstructure of PDF nanoparticles.

The SEM micrograph of *P. daemia* fibre-derived nanoparticles reveals a heterogeneous microstructure characterised by irregularly shaped particles aggregated into dense, compact clusters. The particles exhibit flake-like morphologies with coarse surface textures and non-uniform boundaries, indicating partial agglomeration. This is typical of green-synthesised nanoparticles, where phytoconstituents such as polyphenols, flavonoids, proteins, and organic acids act simultaneously as reducing and stabilising agents. While these biomolecules cap nanoparticle surfaces, steric interactions and weak intermolecular forces often promote localised clustering.^24^ The surface topology is rough and porous, providing a high specific surface area and significant surface energy. Such features enhance interfacial reactivity, making the nanoparticles particularly suitable for antimicrobial and catalytic functions. The porous interparticle networks may facilitate the adsorption of biomolecules, pollutants, or drug molecules, while also enabling improved penetration into microbial biofilms. Size-wise, most primary nanoparticles lie within the tens of nanometres range, though aggregation yields submicron-scale assemblies. The SEM images reveal a highly porous and agglomerated ZnO NP network with abundant interparticle voids and surface cavities at the sub-micron scale. The relatively high apparent porosity (∼30%) arises from loosely packed ZnO nano-crystallites capped by phytochemicals from *P. daemia*, which inhibit dense sintering and promote pore preservation. Such porous morphology enhances surface area, facilitates mass transfer, and improves contact between nanoparticles and bacterial cells, thereby contributing to the observed antibacterial and antibiofilm efficacy. The absence of sharp crystallographic planes or faceted edges corroborates the semi-crystalline nature observed in XRD, where a crystallinity index of 21% was reported. Figure 3 shows the SEM microstructure of PDF nanoparticles. Figure 4 shows the SEM microstructure of PDF nanoparticles.

**Fig. 5:**
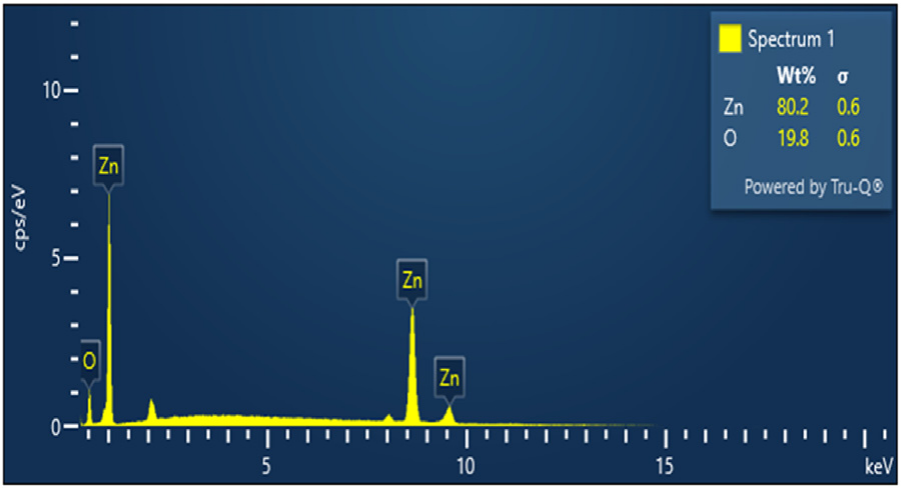
EDX spectrum of PDF nanoparticles.

Energy-dispersive X-ray spectroscopy (EDX) coupled with SEM was employed to confirm the elemental composition of the synthesised nanoparticles (Figure 5). The EDX spectrum exhibited strong and well-defined peaks corresponding exclusively to zinc (Zn) and oxygen (O), confirming the successful formation of ZnO NPs without detectable impurity elements. Quantitative analysis revealed a Zn content of 80.2 wt% and an O content of 19.8 wt%, which is in good agreement with the theoretical stoichiometry of ZnO. The absence of extraneous elemental signals highlights the chemical purity of the nanoparticles and indicates that the phytochemical constituents from the *P. daemia* fibre extract primarily functioned as surface-bound capping agents rather than forming a separate detectable phase. The dominance of Zn peaks further confirms efficient reduction of Zn²⁺ ions and effective nanoparticle crystallisation. Combined with SEM observations, the EDX results substantiate that the synthesised nanostructures are predominantly ZnO with a chemically clean composition, while the observed porous and irregular morphology is expected to enhance surface reactivity and contribute favourably to antibacterial and antibiofilm performance. Future studies will incorporate BET adsorption–desorption analysis to quantify specific surface area, pore volume, and pore size distribution, enabling direct correlation with SEM-observed surface morphology. The SEM and EDX characterisation of *P. daemia* nanoparticles is given in Table.

**Table:** SEM and EDX characterisation of *P. daemia* nanoparticles.

Characterization technique	Observed feature	Elements	Scientific interpretation
SEM – Morphology	Irregular, flake-like particles; compact agglomerates with rough surfaces	Particle size: tens of nm (primary), submicron-scale (aggregates)	Agglomeration attributed to phytochemical residues; roughness and porosity increase surface energy and reactivity
SEM – Surface texture	Porous interparticle networks and irregular grain boundaries	High surface roughness and porosity	Facilitates adsorption, enhances ROS generation, and improves antibacterial and catalytic properties
SEM – Crystallographic features	Absence of distinct lattice planes or sharp facets	Semi-crystalline/amorphous dominant	Consistent with XRD crystallinity index of 21%, phytochemicals restrict lattice propagation
EDX – Major elements	Strong signals of Zn and O	Zn:O ratio ∼1:1 (consistent with ZnO stoichiometry)	Confirms ZnO nanoparticle formation as primary phase
EDX – Minor elements	Weak C signal	Carbon trace from plant-derived capping agents	Organic phytochemicals remain adsorbed, contributing to stabilisation and bioactivity

### Antibacterial activities of *P. daemia* nanoparticles

**Fig. 6:**
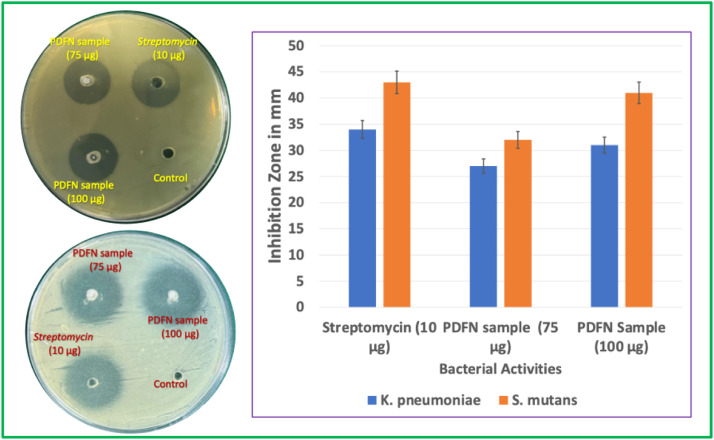
Antibacterial activities of PDF nanoparticles.

The antibacterial activity of *P. daemia* fibre-derived nanoparticles (PDFN) was systematically evaluated against two clinically relevant pathogens, *K pneumoniae* and *S mutans*, using the agar well diffusion assay. The resulting inhibition profiles, presented in Figure 6, clearly demonstrate a concentration-dependent response for both Gram-negative and Gram-positive strains. For *K pneumoniae*, PDFN produced inhibition zones of 27 ± 1 mm at 75 µg and 31 ± 1 mm at 100 µg, indicating enhanced bactericidal performance with increasing nanoparticle concentration. Although streptomycin (10 µg) exhibited a slightly higher inhibition zone of 34 ± 1 mm, the PDFN response at 100 µg approached this benchmark, confirming their considerable efficacy against this opportunistic pathogen known for multidrug resistance. A comparable dose-dependent trend was observed for *S mutans*, a major etiological agent of dental caries and biofilm-mediated oral infections. Streptomycin demonstrated an inhibition zone of 43 ± 1 mm, whereas PDFN produced inhibition zones of 32 ± 1 mm (75 µg) and 41 ± 1 mm (100 µg). The strong response at 100 µg highlights the potential of PDFN as a phytogenic antimicrobial agent capable of inhibiting oral pathogens that contribute significantly to dental plaque formation and enamel demineralisation.

The antibacterial activity of PDFN arises from a synergistic interplay between the ZnO NP core and the phytochemical capping layer inherited from the *P. daemia* extract. ZnO NPs are well-documented for their ability to generate ROS such as superoxide anions, hydroxyl radicals, and hydrogen peroxide. These ROS create oxidative stress that compromises bacterial cell membranes, disrupts intracellular proteins, and damages nucleic acids, ultimately impairing metabolic pathways and replication processes.^25^ The crystalline ZnO lattice promotes electron–hole pair generation under aqueous conditions, further amplifying ROS-mediated bactericidal effects. In parallel, the phytochemical constituents adsorbed onto the nanoparticle surface contribute an additional mode of antibacterial action. Flavonoids, alkaloids, phenols, and terpenoids present in the extract exhibit membrane-disruptive properties, inhibit essential bacterial enzymes, chelate metal ions required for cellular function, and interfere with quorum-sensing mechanisms.^26^ This biochemical interference complements the inorganic ROS-generating action, creating a multi-targeted antimicrobial environment that reduces the likelihood of resistance development. The nanoscale size of PDFN enhances their interaction with bacterial cells by increasing the surface-to-volume ratio, enabling greater contact with the microbial membrane. Their small dimensions facilitate adsorption onto the cell surface, penetration through the peptidoglycan or outer membrane barrier, and accumulation near intracellular targets. With increasing concentration, the number of active surface sites increases, allowing greater ROS flux and higher availability of phytochemical moieties capable of interacting with cellular components. Collectively, these findings confirm the robust antibacterial potential of *P. daemia*-derived ZnO NPs. Their dual-action mechanism, combining inorganic oxidative stress with organic phytochemical disruption, positions PDFN as a potential material for biomedical applications. Potential uses include antimicrobial coatings, wound healing formulations, infection-resistant dental composites, and protective films for healthcare products. The efficacy of PDFN against both Gram-negative and Gram-positive bacteria underscores their relevance for broad-spectrum antimicrobial strategies, particularly in contexts where antibiotic resistance and biofilm formation pose significant clinical challenges.

### Anti-biofilm analysis of *P. daemia* nanoparticles

**Fig. 7:**
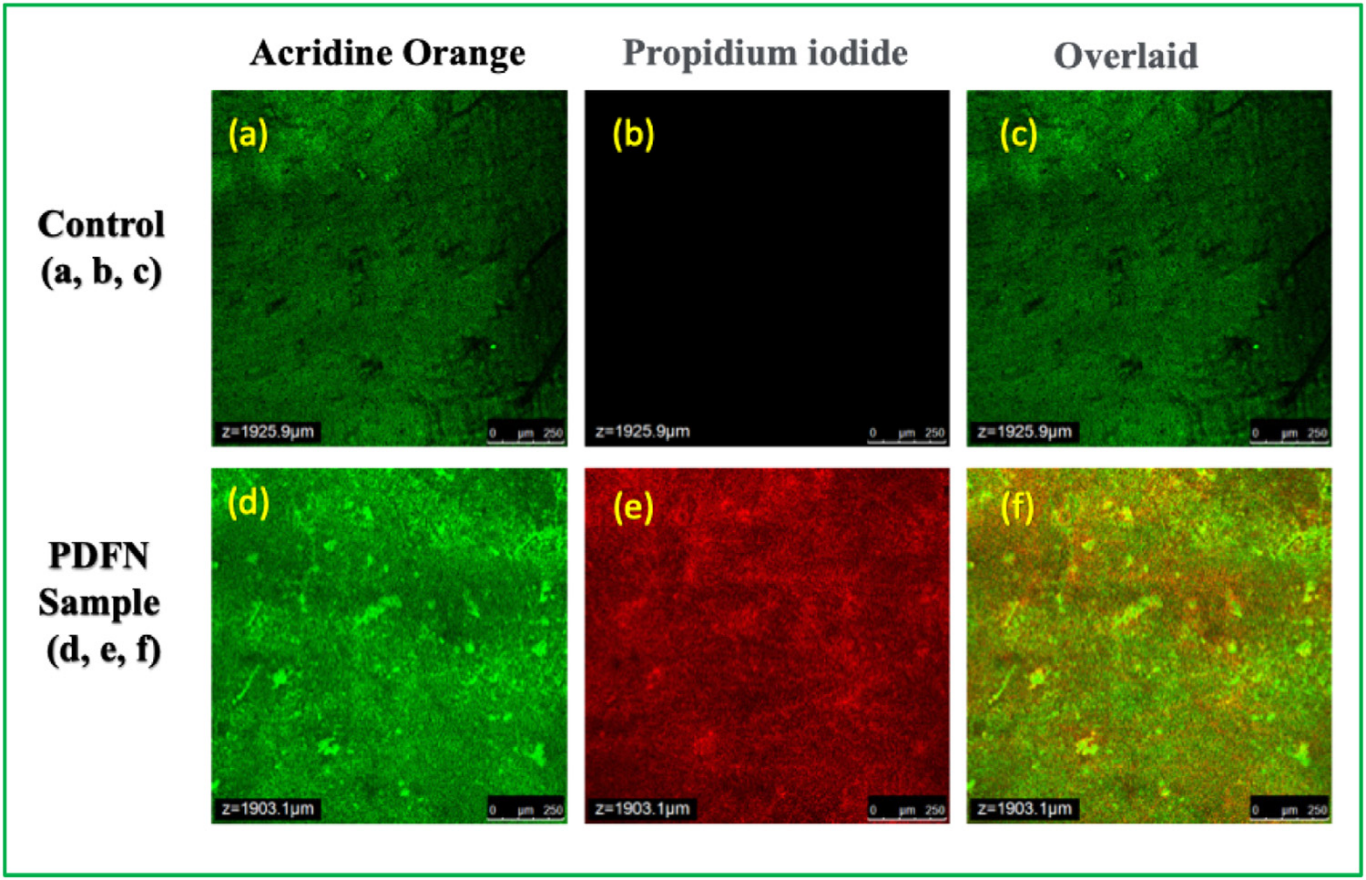
Representative CLSM images showing qualitative biofilm morphology in the presence of PDF nanoparticles.

The antibiofilm activity of the PDF nanoparticles was evaluated using CLSM to visualise biofilm morphology and viability. The CLSM images presented in Figure 7 are representative qualitative observations, illustrating changes in biofilm structure in the presence of the nanoparticles. This analysis was intended to provide visual evidence of biofilm disruption rather than quantitative measurements of biofilm biomass or thickness. Accordingly, the CLSM results are discussed in a qualitative context, and no numerical biofilm parameters were derived from these images. The anti-biofilm efficacy of PDFN was comprehensively assessed using AO and PI dual-fluorescence staining coupled with CLSM. In the untreated control biofilm, CLSM images exhibited predominant green fluorescence from AO, a nucleic acid–binding dye that selectively permeates intact membranes, signifying a high density of metabolically active and viable bacterial populations. The negligible PI signal in the control further confirmed the structural integrity of the cell membranes, demonstrating that bacterial communities were well protected within the extracellular polymeric substance (EPS) and capable of sustained colonisation. Conversely, PDFN treatment resulted in a dramatic shift in fluorescence distribution, where extensive red emission from PI, indicative of compromised membranes and cytoplasmic leakage, was observed. The overlaid micrographs revealed a substantial proportion of dual-coloured (green–red) signals, highlighting the coexistence of partially viable and dead cells, and pointing to a progressive disruption of biofilm integrity under nanoparticle exposure.

The mechanistic basis of this potent antibiofilm effect involves multiple physicochemical interactions. The nanoscale dimension of PDFN allows efficient infiltration into the dense EPS scaffold, a critical barrier that impedes conventional antimicrobials. Once diffused through the biofilm matrix, the nanoparticles interact intimately with bacterial cell envelopes. Electrostatic forces between the negatively charged bacterial surfaces and positively charged or polarizable phytochemical-rich nanoparticle surfaces facilitate adsorption and destabilisation of lipid bilayers. This is further aggravated by the generation of ROS, leading to oxidative stress that damages membrane lipids, denatures intracellular proteins, and induces nucleic acid fragmentation. Such ROS-mediated cytotoxicity compromises cellular homeostasis and accelerates cell death within the biofilm microenvironment.^27^ Also, the phytoconstituents embedded in PDFN, such as flavonoids, alkaloids, and phenolic derivatives, likely exert synergistic antimicrobial action by inhibiting metabolic enzymes and disrupting electron transport chains. Beyond direct cytotoxicity, PDFN appears to impair the collective resilience of the biofilm. By penetrating and destabilising the EPS, nanoparticles weaken the cohesive viscoelastic properties of the matrix, rendering the microbial community more vulnerable to environmental stresses. Moreover, interference with quorum-sensing (QS) regulatory pathways is a plausible mechanism,^28^ where PDFN may disrupt autoinducer signalling molecules, thereby attenuating intercellular communication essential for biofilm maturation, virulence factor production, and persistence. The combination of EPS disintegration, ROS-induced cellular damage, and quorum-quenching culminates in a significant reduction in viable biofilm biomass, as confirmed by the AO/PI staining pattern. Taken together, the data demonstrate that *P. daemia* nanoparticles exhibit a multifaceted antibiofilm mechanism, simultaneously targeting bacterial membranes, intracellular metabolism, extracellular scaffolds, and communication pathways. Such a broad-spectrum inhibitory profile positions PDFN as a promising nanomaterial for advanced biomedical and dental applications, including implant coatings, restorative composites, wound dressings, and bio-surface modifications, where biofilm suppression and long-term antimicrobial functionality are critical for clinical success.

### Thermal stability of *P. daemia* nanoparticles

**Fig. 8:**
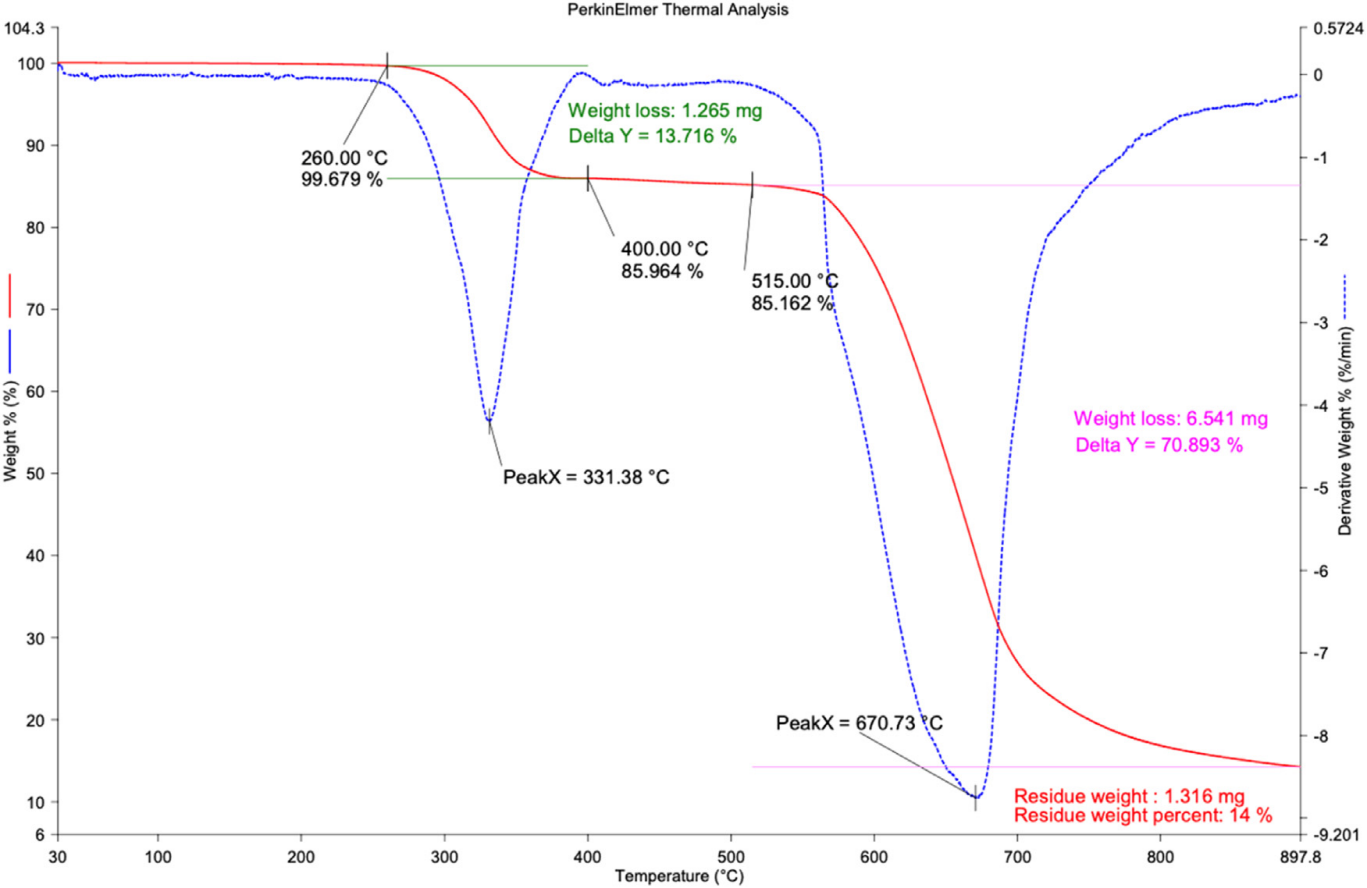
TGA/DTG curves of PDF nanoparticles.

The thermogravimetric (TGA) and derivative thermogravimetric (DTG) thermograms of *P. daemia* nanoparticles elucidate a multiphase thermal decomposition sequence, reflecting the heterogeneous composition of biogenically synthesised nanomaterials. The initial thermal regime up to 260 °C exhibits only a trivial mass decline of about 0.32%, attributed primarily to the desorption of physiosorbed water molecules, entrapped volatiles, and removal of loosely bound phytochemicals. This stage signifies that the nanoparticles exhibit pronounced thermal inertia in the sub-ambient and moderate heating range, suggesting their feasibility for incorporation into polymer matrices processed below 250 °C without risk of premature degradation. The first substantive weight-loss event, occurring between 260 and 400 °C, is marked by a DTG maximum at 331.38 °C, accounting for a reduction of 1.265 mg (13.716%). The mechanistic basis of this decomposition can be ascribed to the scission of weak glycosidic linkages in hemi-cellulosic constituents, depolymerisation of amorphous cellulose, cleavage of esterified side groups, and volatilisation of low-molecular-weight oligomers.^29^ In this stage, degradation proceeds via dehydration, decarboxylation, and depolymerisation pathways, facilitated by the presence of oxygenated moieties such as hydroxyl, carbonyl, and carboxylate groups, which destabilise the molecular backbone.^30^ The second dominant decomposition regime spans 400 to 670 °C and is characterised by a sharp DTG inflexion at 670.73 °C, corresponding to a significant weight loss of 6.541 mg (70.893%). This stage reflects the thermochemical decomposition of crystalline cellulose domains, fragmentation of lignin aromatics, and oxidative cleavage of high-molecular-weight biopolymeric chains. The DTG profile’s steepness indicates a rapid pyrolytic degradation mechanism dominated by glycosidic bond rupture, demethoxylation of lignin units, and volatilisation of aromatic hydrocarbons.^31^ Moreover, this region coincides with oxidative char fragmentation and devolatilization, which accelerates mass loss. The thermal resilience observed up to this zone underscores the semi-crystalline architecture and robust lignocellulosic framework of the nanoparticles. Post-670 °C, the thermal curve plateaus, with a residual char fraction of about 1.316 mg (14% of the initial mass). The persistence of this residue indicates the formation of carbonaceous char enriched with thermally inert inorganic phases, likely consisting of oxides, silicates, or mineral salts inherently present in the phytogenic precursor. The stabilisation of residue at elevated temperatures suggests the retention of refractory compounds capable of enhancing thermal shielding and flame-retardant properties when incorporated into composite systems.^32^ Mechanistically, the overall degradation pathway of *P. daemia* nanoparticles involves moisture volatilization and desorption below 260 °C, depolymerization of amorphous carbohydrate fractions and functional group scission between 260 and 400 °C, decomposition of crystalline cellulose, lignin, and aromatic phases via bond cleavage, demethoxylation, and oxidative pyrolysis between 400 and 670 °C, followed by carbonization and stabilization of inorganic char beyond 670 °C. This multi-step thermal behaviour highlights the synergistic presence of organic biomacromolecules and inorganic stabilising residues, which confer both degradability and high-temperature resilience. Compared to other plant-derived nanoparticulates such as jute, flax, or banana fibre, the elevated degradation maxima above 670 °C and substantial char yield of 14% underline the superior thermo-oxidative resistance and residue-forming capacity of *P. daemia* nanoparticles. Such properties are advantageous for biopolymer reinforcement, flame-retardant coatings, and biomedical systems, where controlled degradation and thermal endurance are vital. Figure 8 shows the TGA/DTG curves of PDF nanoparticles.

## Conclusion

This study demonstrates the green synthesis of ZnO NPs from *P. daemia* fibre extracts via a sustainable, plant-mediated approach. Comprehensive structural, morphological, thermal, and biological analyses confirmed the formation of stable, phytochemical-functionalized nanoparticles with nanoscale dimensions and multifunctional properties. FTIR spectroscopy identified hydroxyl, carbonyl, and carboxylate groups as key reducing and stabilising agents, while SEM revealed irregular, porous nanoscale morphologies conducive to biological interactions. XRD confirmed a semi-crystalline structure with nanosized crystallites, and TGA established appreciable thermal stability with multi-step degradation and ∼14% residual char.

Biological evaluations highlighted strong antibacterial and antibiofilm activities, underscoring the translational potential of these nanoparticles in dentistry. In line with previous reports on ZnO NPs inhibiting *S mutans* biofilms, enhancing root canal disinfection, and improving antimicrobial protection in restorative and implant coatings, the present findings suggest that *P. daemia*-derived ZnO NPs can serve as natural and sustainable alternatives to conventional chemical agents and antibiotics.

Beyond oral health care, the valorisation of *P. daemia* fibres contributes to waste-to-resource conversion and aligns with circular bioeconomy principles. Around 60% to 70% of the biomass-based process can be directed towards high-value applications, including antimicrobial agents, eco-friendly biomedical coatings, drug delivery platforms, and polymer composites. Thus, *P. daemia*-derived ZnO NPs represent a multifunctional nanomaterial platform of dual importance advancing infection control in dentistry while fostering sustainable material innovation.

### Limitations and future work

This study confirms the antibacterial activity of *P. daemia*-derived ZnO NPs, but several limitations remain. Key physicochemical parameters such as optical bandgap, zeta potential, and long-term stability were not evaluated, and only two bacterial strains were tested. Quantitative assays (MIC/MBC, time–kill studies) and cytotoxicity analyses were not performed, limiting insight into dose–response relationships and biocompatibility.

Future work will include UV–Vis analysis with Tauc plots, surface charge measurements, and stability assessments to better understand ROS-related mechanisms. Antimicrobial testing will be expanded to additional Gram-positive, Gram-negative, and drug-resistant strains, along with MIC/MBC evaluations and biofilm quantification. Cytocompatibility studies using mammalian cells and preliminary integration of the nanoparticles into dental and biomedical materials will also be explored to support translational potential.

## Author contributions

*Conceptualisation, methodology, data curation, formal analysis, writing – original draft*: Thandavamoorthy; *Supervision, visualisation, writing – review & editing, project administration, corresponding author:* Devarajan; *Validation, resources, critical revision of the manuscript, and funding acquisition*: Mehar.

## Declaration of generative AI and AI-assisted technologies in the writing process

The authors acknowledge the use of AI-assisted tools, notably Grammarly, to improve linguistic accuracy, correct grammatical errors, and increase the overall coherence of this article. The content, analytical discussion, and conclusions presented in this work are distinctly the original contributions of the author, with AI tools utilised solely to enhance the presentation and clarity of the text. The use of AI complies with the journal's requirements for transparency and ethical norms in authorship processes.

## Funding

This research did not receive any specific grant from funding agencies in the public, commercial, or not-for-profit sectors.

## Conflict of interest

None disclosed.
